# Positive and negative influences of social participation on physical and mental health among community-dwelling elderly aged 65–70 years: a cross-sectional study in Japan

**DOI:** 10.1186/s12877-017-0502-8

**Published:** 2017-05-19

**Authors:** Kimiko Tomioka, Norio Kurumatani, Hiroshi Hosoi

**Affiliations:** Nara Prefectural Health Research Center, Nara Medical University, Shijo-cho 840, Kashihara city, Nara, 634-8521 Japan

**Keywords:** Social participation, Mental health, Physical health, Autonomy, Gender differences, Elderly

## Abstract

**Background:**

Although numerous investigations have indicated that social participation (SP) has positive effects on the health of older adults, there have been few studies on its negative health consequences. We examined the cross-sectional associations of the type, frequency, and autonomy for SP with physical and mental health.

**Methods:**

The analytical subjects were 5126 males and 7006 females who were functionally independent, born between 1945 and 1949, and covered by A City’s medical insurance system. Physical and mental health were measured using the SF-8 Health Survey. SP was measured through six types of social groups. These social groups included volunteer groups, sports groups, hobby clubs, senior citizens’ clubs, neighborhood community associations, and cultural groups. Analysis of covariance was conducted to compare adjusted physical health component summary scores (PCS) and mental health component summary scores (MCS) by the frequency and autonomy of SP. Age, family size, body mass index, chronic conditions, smoking, alcohol intake, depression and cognitive functioning were included as covariates. To examine whether the associations between SP and PCS/MCS are different between genders, we performed analyses stratified by gender.

**Results:**

Overall, positive associations of the frequency and autonomy of SP with PCS and MCS were stronger in females than males. As to frequency, frequent participation in sports groups and hobby clubs had significantly better PCS among both genders and better MCS among females than non-participation. None of the groups differed significantly in the MCS among males. As to autonomy, among both genders, voluntary participation in sports groups and hobby clubs had significantly better PCS than non-participation, and better MCS than not only non-participation, but also obligatory participation. Among females, obligatory participation in all groups had significantly poorer MCS than voluntary participation, and obligatory participation in sports groups had significantly poorer MCS than non-participation.

**Conclusions:**

Obligatory SP had significantly poorer MCS than voluntary participation, occasionally than non-participation; there is a possibility that obligatory SP has harmful influences on mental health of community-dwelling elderly. Measures to promote SP with consideration for individuals’ autonomy may be effective in the public health approach to maintaining mental health.

## Background

Social participation (SP) is a determinant of active aging [1], and there is a great deal of evidence that SP can produce positive health outcomes, in both physical health [2, 3] and mental health [4–6]. The mechanisms of how elderly people’s SP benefits their health are considered as follows. 1) SP encourages physical activity, allowing participants to maintain physical function (i.e., the “use-it-or-lose-it” hypothesis) [3]. 2) Social support and networks gained through SP provide information that helps participants make better health and medical choices. Therefore, those participants have healthier lifestyles and they can maintain good physical and mental health [7, 8]. 3) SP provides positive psychological states including better self-esteem, a sense of belonging, and a purpose in life for those who participate. Positive psychological states have a strong impact on mental health [6, 7, 9], and have a protective effect against a decline in physical function [10]. 4) Social support and networks derived from SP play a role in alleviating stress in participants, preventing them from developing functional decline [11] and mental health problems that come from stress (i.e., stress-buffering effect) [8, 9].

However, it has been pointed out that SP is not only beneficial, but also has a dark side [12–14]. Mitchell et al. [15] have found that SP is positively associated with mental distress, and suggested that it may cause additional obligations in an already stressful daily life, resulting in a negative association between SP and mental health. Iwase et al. [16] have reported that participation in parents and teachers associations is not associated with better self-rated health, and pointed out that the obligatory participation which characterizes this activity may offset its health benefits. These prior studies have suggested that whether SP induces an obligation, or if people participate in social groups without being forced to, is a key point of SP health effects. Therefore, it is indispensable to examine the relationship between SP and the health of elderly people, focusing on the individuals’ autonomy.

Moreover, some prior studies have suggested that frequent SP does not always have a positive health effect on older people. Takeuchi et al. [2] showed that frequent participation in sports groups and hobby clubs was associated with better dental health, but there was a significant association between infrequent participation in neighborhood community associations and better dental health. Musick et al. [17] reported that vigorous volunteering had no protective effect on mortality, and moderate volunteering was associated with a lower risk for mortality. These studies have suggested that frequent participation in a specific type of SP has the potential to produce a sense of obligation to participate, which may not have a beneficial effect on their health. These suggestions show that the type and frequency of SP is an important evaluation indicator.

As for gender differences in the health effects of SP, SP may harm the mental health of females more than males, because females tend to value emotional relationships more than males and more readily suffer psychological distress by being involved with issues derived from people outside their family with whom they have built emotional ties [13]. However, some studies have reported that SP yields greater benefits on physical function [3] and mental health [5, 18] in females than in males. Moreover, a prior study has reported that males are highly influenced by support from their spouse, while females are significantly impacted by support from relatives and friends [19]. Because SP can provide social support from people other than their spouse, females can potentially receive not only more benefits but also more harm from SP than males.

In this study, to unravel whether SP has different influences on the physical and mental aspects of elderly people’s health, whether it has negative aspects, and whether those influences are different between genders, we investigated the cross-sectional relationships of the type and frequency of SP, and autonomy of individuals for SP on their physical and mental health according to gender.

## Methods

### Participants

We used data from the Nara Data Health Survey, a cross-sectional, population-based survey of baby boomers [20]. In 2015, the oldest Japanese baby boomers reached the age of 65, and made up a large proportion of the elderly population. In the ultra-super aging society, the baby boomers are expected to play a lively part in social participation after retirement from active work. In October 2015, city government officers mailed self-administered questionnaires to 19,354 citizens who were born in the years 1945–1949 and covered by the medical insurance system administered by A City in Nara prefecture. Those 19,354 residents to whom we sent the questionnaire comprised approximately 65% of all citizens in the city who were born from 1945 to 1949 as of January 1, 2015. The response rate was 65.9% (*n* = 12,747). Subjects with missing data on physical and mental health (*n* = 189), basic activities of daily living (BADL) (*n* = 161), and all SP questions (*n* = 51) were excluded. Subjects with dependent BADL were also excluded (*n* = 214), because BADL disabilities are an obstacle to SP [21]. Dependent BADL is defined as subjects who needed any assistance with at least one of five BADL items: eating, dressing, bathing, going to the bathroom, and walking indoors.

### Physical and mental health

Physical and mental health were assessed using the Japanese version of the 8-item Short-Form Health Survey (SF-8) [22]. The SF-8 consists of the following 8 subscales: general health (GH), physical functioning (PF), role-physical (RP), bodily pain (BP), vitality (VT), social functioning (SF), mental health (MH), and role-emotional (RE). The SF-8 is the most recent shortened version of the SF-36 [23], and its validity and reliability have been established [22, 24]. Additionally, from the 8 subscale scores, the SF-8 can calculate two summary scores, namely, the Physical Health Component Summary Score (PCS) for physical health and the Mental Health Component Summary Score (MCS) for mental health. We calculated the PCS and MCS scores based on the manual of the Japanese version of the SF-8 [22]: the general Japanese population has a mean score of 50 and a standard deviation of 10, PCS = 0.23024*GH + 0.40672*PF + 0.38317*RP + 0.33295*BP + 0.07537*VT + (−0.01275)*SF + (−0.30469)*MH + (−0.14803)*RE + 0.67371, and MCS = (−0.02020)*GH + (−0.19972)*PF + (−0.16579)*RP + (−0.15992)*BP + 0.16737*VT + 0.27264*SF + 0.57583*MH + 0.42927*RE + 4.34744. Higher scores indicate better health status.

### Social participation

Based on prior studies [2–5, 16, 20], SP was classified into six groups: volunteer groups, sports groups, hobby clubs, senior citizens’ clubs, neighborhood community associations, and cultural groups. Subjects were asked about their frequency of participation in each group: ≥4 times a week, several times a week, once a week, several times a month, several times a year, or non-participation. Because there were few persons with several times or more a week, the frequency of SP was re-categorized into once or more a week (i.e., weekly or more), several times a month (i.e., monthly), several times a year (i.e., yearly), and non-participation. Regarding senior citizens’ clubs, because non-participation accounted for about 90% of analytical subjects, the frequency of participation in senior citizens’ clubs was re-grouped into monthly or more, yearly, and non-participation. Additionally, subjects were asked about their autonomy of participation in each group: very voluntary, rather voluntary, rather obligatory, very obligatory, and non-participation. Because few participants responded with rather obligatory or very obligatory, the autonomy of SP was re-categorized into very voluntary, rather voluntary, very/rather obligatory (i.e., obligatory participation), and non-participation. Moreover, we calculated the number of groups in which the subjects were engaged as a comprehensive indicator of SP. What follows are definitions of each group and their features in Japan:

Volunteer groups: Participants provide voluntary service without payment. Their activities include supporting disaster victims, children, aged people, and disabled people, cleaning activities, disaster mitigation activities, and fund-raising. In Japan, even when participation is not voluntary, if the activities are conducted without receiving payment, it is considered a volunteer activity. For gender difference, males tend to join volunteer activities after retirement, while females are more apt to participate in them during their child-raising years [25].

Sports groups: Participants do sport-related activities. For aged people, they are not competitive but activities in which people can participate easily, such as stretching calisthenics, gateball, and ground golf. Also, many aged people participate in walking events regularly planned by municipal branches of the Japan Walking Association [26] with their friends to visit popular venues. For gender difference, males lean toward more vigorous sports, while females tend to like mild ones.

Hobby clubs: Participants join hobby type activities including fancywork, ceramics, technical art, painting, and group singing. In Japan, tea ceremony, flower arrangement and calligraphy are three traditional performing arts enjoyed by people of all ages as a hobby. For gender difference, ceramics and technical art are popular among males, while fancywork, tea ceremony, and flower arrangement are prevalent in females.

Senior citizens’ clubs: According to the Japan Federation of Senior Citizens Clubs [27], a senior citizens club is an aged people’s autonomously conducted organization based in their community. As of the end of March, 2016, there are 110,000 such clubs with 6.7 million participants. Senior citizens’ clubs conduct various activities for aged people to help give meaning to their lives, improve their physical conditions, and offer friendship by visiting those who live alone or are bed-ridden. Group members must be 60 or older and the groups must receive support from the national or local government. Community-dwelling elderly who live alone are frequently invited to participate in senior citizens’ clubs. Because the husband is usually older than the wife and males have a shorter life-span than females, most solitary aged persons are female.

Neighborhood community associations: Groups that hold activities to expand neighborhood cooperation and make their communities better places to live. These include fire prevention activities, traffic safety, information sharing, community beautification, crime prevention, and neighborhood exchange. Neighborhood community associations are voluntary groups and participation is arbitrary, but often all households in the area participate. Generally, females, especially full-time housewives, play central roles in the daily activities of neighborhood community associations, while males, especially those who have retired from their job, occupy the presidencies of neighborhood community associations.

Cultural groups: Since people’s interest in lifelong learning has increased in Japan, there are a lot of reading clubs, study classes, and citizen’s college lectures in the fields of literature, history, politics, education, women’s issues, and so on. There are college classes for people who are 65 or older. Citizen’s college/Aged people’s college classes are conducted by local governments, but some universities also provide such classes. For gender difference, females are more likely to attend cultural groups regarding social issues (e.g., environmental pollution and human rights problems) than males.

### Covariates

Information on age, gender, and medical history was offered by the city government, and other covariates were gathered from the self-administered questionnaire. Age as of 1st October 2015 was dichotomized into 65–67 years and 68–70 years. Family size was categorized as one (i.e., living alone), two, three, and four or more persons. Body mass index (BMI) was categorized as normal (i.e., 18.5- < 25.0), thin (i.e., <18.5), or overweight (i.e., ≥25.0). For current medical history, the names of the following diseases diagnosed by attending physician were extracted from health insurance claims; hypertension, diabetes mellitus, hyperlipidemia, cerebrovascular disease, heart disease, and renal disease. The number of comorbidities was categorized as 0, 1, 2, or ≥3. Smoking was classified as never-smoker, ex-smoker, or current smoker. Alcohol intake was classified as none, social, occasional, or daily drinker. Frequency of fitness habits (hours per week) was classified as <1, 1–2, 3–4, or ≥5. Depression was measured using the 5-item short form of the Geriatric Depression Scale (score range 0–5) [28]. Participants whose score was ≥2 were defined as having depression. Cognitive functioning was measured with the Cognitive Performance Scale (score range 0–6) [29]. Individuals whose score was ≥1 were defined as having poor cognitive functioning.

### Statistical analysis

The general linear model was used to assess the associations of the type, frequency, and autonomy for SP with the PCS and the MCS. Adjusted mean scores for the PCS and the MCS were calculated using the analysis of covariance (ANCOVA). Multiple comparisons were conducted using a Bonferroni-adjusted test of significance. As to multicollinearity between independent variables and anticipated covariates, a moderately high correlation between participation in sports groups and fitness habits (Spearman’s coefficient of 0.57, *p* < 0.001) was identified. Therefore, we decided to eliminate fitness habits from the covariates. Finally, covariates included in the adjustment were age, family size, BMI, comorbidities, smoking, alcohol intake, depression and cognitive functioning. To deal with missing covariate data, we used multiple imputation by chained equation routines implemented in IBM SPSS Missing Value Version 24 [30]. To examine whether the associations of SP with the PCS and MCS are different between genders, we performed analyses stratified by gender. Statistical analyses were performed using IBM SPSS Statistics (version 24.0 J, IBM SPSS Inc., Chicago, USA). Significance was set at *p* < 0.05 (two-sided test).

## Results

We excluded 615 subjects based on the exclusion criteria, and identified 12,132 participants with independent BADL (5126 males, 7006 females) as analytical subjects. Table 1 shows the crude mean PCS and MCS according to covariates among the study participants. There was no gender difference in the PCS, but females were more likely to have poorer MCS than males. People aged 68–70 were more likely to have poorer PCS than people aged 65–67, but there was no age difference in the MCS. Those living alone, those with three or more chronic diseases, current smokers, non-drinkers, those with depression, and those with poor cognitive functioning had the worst PCS and MCS scores. Regarding BMI, the worst scores were observed in overweight people for the PCS, but in thin people for the MCS.

**Table 1:** Crude mean (standard deviation) for physical and mental health component summary scores among the study population, by covariates (*n* = 12,132)

	n	Physical Health Component Summary								Mental Health Component Summary								
		Mean (SD)							*P* value	Mean (SD)								*P* value
Gender																		
Male	5,126	49.08 (6.69)							0.731^a^	51.17 (6.00)								<0.001^a^
Female	7,006	49.13 (6.68)								50.58 (6.00)								
Age at survey																		
65-67	5,798	49.27 (6.52)							0.012^a^	50.75 (5.97)								0.178^a^
68-70	6,334	48.96 (6.82)								50.90 (6.04)								
Family size																		
1	1,775	48.41 (7.24)	*	*	*				<0.001^b^	49.93 (6.65)	*	*	*	*				<0.001^b^
2	6,291	49.29 (6.49)	*							51.12 (5.86)	*							
3	2,493	49.10 (6.69)		*						50.76 (5.92)		*						
≥4	1,478	49.14 (6.76)			*					50.71 (5.92)			*					
Missing	95	49.72 (6.46)								51.70 (4.64)				*				
Body mass index																		
Normal	8,798	49.63 (6.31)	*	*					<0.001^b^	50.91 (5.91)	*							<0.001^b^
Thin	1,009	49.02 (6.89)	*		*					49.75 (6.62)	*	*						
Overweight	2,225	47.09 (7.56)		*	*	*				51.02 (6.04)		*						
Missing	100	48.98 (7.17)				*				50.10 (6.22)								
Number of chronic diseases under medical treatment																		
None	4,852	50.17 (6.04)	*	*	*				<0.001^b^	51.18 (5.55)	*	*	*					<0.001^b^
1	2,716	49.27 (6.41)	*			*	*			50.55 (6.34)	*							
2	2,515	48.47 (6.89)		*		*		*		50.78 (5.97)		*						
≥3	2,049	47.16 (7.65)			*		*	*		50.43 (6.57)			*					
Smoking																		
Never-smoker	6,809	49.31 (6.54)	*	*					0.003^b^	50.86 (5.87)	*							0.002^b^
Ex-smoker	3,551	48.90 (6.72)	*							50.99 (5.95)		*						
Current smoker	1,555	48.79 (7.13)		*						50.30 (6.76)	*	*						
Missing	217	48.62 (6.94)								50.93 (5.18)								
Frequency of alcohol consumption																		
None	4,737	48.53 (7.15)	*	*	*				<0.001^b^	50.41 (6.35)	*	*	*					<0.001^b^
Social	2,679	49.26 (6.60)	*							50.87 (5.73)	*			*	*			
Occasional	1,890	49.56 (6.23)		*						51.10 (5.61)		*				*		
Daily	2,768	49.64 (6.13)			*					51.36 (5.85)			*	*			*	
Missing	58	49.05 (7.03)								48.59 (6.69)					*	*	*	
Depression																		
Absent	9,338	49.89 (5.98)	*	*					<0.001^b^	51.97 (4.92)	*	*						<0.001^b^
Present	2,577	46.31 (8.17)	*		*					46.69 (7.58)	*		*					
Missing	217	48.57 (6.56)		*	*					50.65 (5.93)		*	*					
Cognitive functioning																		
Intact	10,587	49.57 (6.38)	*						<0.001^b^	51.28 (5.61)	*							<0.001^b^
Poor	1,524	45.94 (7.80)	*							47.68 (7.50)	*							
Missing	21	48.64 (7.10)								49.37 (9.39)								

^a^Differences between two groups were analyzed using the t test

^b^Differences between three or more groups were analyzed using one-way analysis of variance (ANOVA)

^*^ *P*<0.05 with multiple comparison test by the Tukey method

Table 2 shows the results on the association between the frequency of SP and physical health. Among both genders, the frequency of participation in sports groups and hobby clubs differed significantly in the PCS. A multiple comparison test revealed that frequent participation (i.e., weekly or more) had significantly better PCS compared with non-participation. Additionally, among females, the frequency of participation in volunteer groups, neighborhood community associations, and cultural groups differed significantly in the PCS, but there were no significant differences in the PCS between frequent participation and non-participation.

**Table 2:** Adjusted mean (standard error) for the Physical Health Component Summary Score for the type and frequency of social participation, stratified by gender

	Males					Females					Interaction
	N^a^	Mean (SE)			*P* ^b^	N^a^	Mean (SE)			*P* ^b^	
Volunteer groups											
Non-participation	4,151	46.69 (0.20)			0.708	5,723	46.19 (0.20)	*		0.004	*p* = 0.244
Yearly	334	46.58 (0.40)				451	46.77 (0.35)				
Monthly	346	46.78 (0.39)				487	46.98 (0.35)	*			
Weekly or more	204	47.19 (0.48)				222	47.10 (0.47)				
Sports groups											
Non-participation	3,702	46.58 (0.20)	*		0.001	4,713	46.05 (0.20)	*	*	<0.001	*p* = 0.934
Yearly	290	46.99 (0.42)				152	46.63 (0.54)				
Monthly	487	47.33 (0.35)				651	47.06 (0.32)	*			
Weekly or more	555	47.59 (0.34)	*			1,353	47.14 (0.26)		*		
Hobby clubs											
Non-participation	3,352	46.57 (0.20)	*	*	0.002	3,754	46.03 (0.20)	*	*	<0.001	*p* = 0.844
Yearly	504	46.44 (0.34)				511	46.23 (0.34)				
Monthly	826	47.26 (0.29)	*			1,832	46.82 (0.25)	*			
Weekly or more	350	47.57 (0.39)		*		789	46.92 (0.30)		*		
Senior citizens’ clubs											
Non-participation	4,596	46.74 (0.20)			0.115	6,407	46.27 (0.20)			0.932	*p* = 0.531
Yearly	311	46.04 (0.41)				338	46.17 (0.39)				
Monthly or more	141	46.18 (0.57)				137	46.13 (0.57)				
Neighborhood community associations											
Non-participation	2,988	46.66 (0.20)			0.793	3,914	46.07 (0.21)	*	*	<0.001	*p* = 0.029
Yearly	1,431	46.82 (0.25)				2,370	46.51 (0.23)	*			
Monthly	484	46.55 (0.35)				414	47.33 (0.36)		*		
Weekly or more	112	46.88 (0.63)				78	46.11 (0.74)				
Cultural groups											
Non-participation	4,405	46.67 (0.20)			0.366	5,436	46.19 (0.20)			0.010	*p* = 0.553
Yearly	261	46.93 (0.44)				490	46.84 (0.35)				
Monthly	273	46.56 (0.43)				718	46.74 (0.31)				
Weekly or more	101	47.72 (0.66)				218	47.04 (0.47)				

The data in this table are adjusted for age, family size, BMI, comorbidities, smoking, alcohol intake, depression and cognitive functioning

^a^The total N varies slightly between each group as a result of missing data

^b^This is by use of an analysis of covariance (ANCOVA) for differences in mean scores

^*^ *P*<0.05 with the Bonferroni correction method

Table 3 shows the results on the association between the frequency of SP and mental health. Among males, all groups had no association between the frequency of SP and the MCS. Among females, frequency of participation in sports groups, hobby clubs, neighborhood community associations, and cultural groups was significantly associated with the MCS. Frequent participation in sports groups and hobby clubs had significantly better MCS compared with non-participation. Frequent participation in cultural groups had significantly better MCS than infrequent participation (i.e., yearly), but had no difference in MCS from non-participation. Regarding neighborhood community associations, only infrequent participation had significantly better MCS than non-participation.

**Table 3:** Adjusted mean (standard error) for the Mental Health Component Summary Score for the type and frequency of social participation, stratified by gender

	Males			Females					Interaction
	N^a^	Mean (SE)	*P* ^b^	N^a^	Mean (SE)			*P* ^b^	
Volunteer groups									
Non-participation	4,151	48.44 (0.17)	0.582	5,723	47.62 (0.18)			0.064	*p* = 0.243
Yearly	334	48.30 (0.34)		451	48.02 (0.31)				
Monthly	346	48.19 (0.34)		487	48.12 (0.31)				
Weekly or more	204	48.82 (0.42)		222	48.23 (0.41)				
Sports groups									
Non-participation	3,702	48.33 (0.17)	0.078	4,713	47.56 (0.18)	*		<0.001	*p* = 0.682
Yearly	290	48.57 (0.37)		152	47.43 (0.48)				
Monthly	487	48.88 (0.30)		651	48.02 (0.28)				
Weekly or more	555	48.79 (0.29)		1,353	48.28 (0.23)	*			
Hobby clubs									
Non-participation	3,352	48.38 (0.17)	0.467	3,754	47.49 (0.18)	*	*	<0.001	*p* = 0.383
Yearly	504	48.46 (0.29)		511	47.90 (0.30)				
Monthly	826	48.44 (0.25)		1,832	47.94 (0.22)	*			
Weekly or more	350	48.88 (0.34)		789	48.55 (0.26)		*		
Senior citizens’ clubs									
Non-participation	4,596	48.41 (0.17)	0.444	6,407	47.65 (0.17)			0.089	*p* = 0.596
Yearly	311	48.21 (0.35)		338	47.90 (0.34)				
Monthly or more	141	48.92 (0.49)		137	48.65 (0.50)				
Neighborhood community associations									
Non-participation	2,988	48.39 (0.17)	0.554	3,914	47.53 (0.18)	*		0.009	*p* = 0.108
Yearly	1,431	48.37 (0.21)		2,370	48.01 (0.20)	*			
Monthly	484	48.76 (0.30)		414	47.63 (0.32)				
Weekly or more	112	48.34 (0.54)		78	48.25 (0.65)				
Cultural groups									
Non-participation	4,405	48.41 (0.17)	0.968	5,436	47.65 (0.18)			0.001	*p* = 0.071
Yearly	261	48.49 (0.38)		490	47.17 (0.31)	*	*		
Monthly	273	48.35 (0.37)		718	48.23 (0.27)	*			
Weekly or more	101	48.20 (0.57)		218	48.58 (0.41)		*		

The data in this table are adjusted for age, family size, BMI, comorbidities, smoking, alcohol intake, depression and cognitive functioning

^a^The total N varies slightly between each group as a result of missing data

^b^This is by use of an analysis of covariance (ANCOVA) for differences in mean scores

^*^ *P*<0.05 with the Bonferroni correction method

Table 4 shows the results on the association between the autonomy of SP and physical health. Among both genders, the autonomy of participation in sports groups and hobby clubs differed significantly in the PCS. Very voluntary participation in both groups had significantly better PCS than non-participation. Additionally, among females, the autonomy of participation in volunteer groups, neighborhood community associations, and cultural groups differed significantly in the PCS, but significant difference between very voluntary participation and non-participation was observed only in cultural groups. Obligatory participation in neighborhood community associations had significantly better PCS than non-participation.

**Table 4:** Adjusted mean (standard error) for the Physical Health Component Summary Score for the type and autonomy of social participation, stratified by gender

	Males					Females					Interaction
	N^a^	Mean (SE)			*P* ^b^	N^a^	Mean (SE)			*P* ^b^	
Volunteer groups											
Non-participation	3,995	46.66 (0.20)			0.430	5,568	46.17 (0.20)	*		0.004	*p* = 0.421
Very/rather obligatory	341	46.93 (0.39)				298	46.11 (0.41)				
Rather voluntary	379	46.84 (0.38)				567	47.06 (0.33)	*			
Very voluntary	372	47.19 (0.38)				522	46.76 (0.34)				
Sports groups											
Non-participation	3,627	46.59 (0.20)	*		<0.001	4,654	46.02 (0.20)	*	*	<0.001	*p* = 0.650
Very/rather obligatory	144	46.42 (0.56)				158	46.56 (0.53)				
Rather voluntary	493	47.21 (0.34)				757	46.97 (0.30)	*			
Very voluntary	811	47.93 (0.30)	*			1,361	47.10 (0.26)		*		
Hobby clubs											
Non-participation	3,230	46.58 (0.20)	*		<0.001	3,674	46.02 (0.20)	*	*	<0.001	*p* = 0.190
Very/rather obligatory	144	46.14 (0.56)				173	46.68 (0.51)				
Rather voluntary	720	46.64 (0.30)		*		1,122	46.68 (0.27)	*			
Very voluntary	983	47.63 (0.28)	*	*		1,962	46.97 (0.25)		*		
Senior citizens’ clubs											
Non-participation	4,531	46.75 (0.20)			0.317	6,350	46.27 (0.20)			0.351	*p* = 0.240
Very/rather obligatory	298	46.64 (0.41)				302	45.73 (0.41)				
Rather voluntary	175	45.87 (0.51)				204	46.72 (0.48)				
Very voluntary	85	47.07 (0.72)				91	46.22 (0.69)				
Neighborhood community associations											
Non-participation	2,557	46.60 (0.21)			0.251	3,169	45.92 (0.21)	*	*	<0.001	*p* = 0.014
Very/rather obligatory	1,692	46.82 (0.24)				2,623	46.66 (0.22)	*			
Rather voluntary	566	46.80 (0.33)				869	47.16 (0.29)		*		
Very voluntary	257	47.38 (0.44)				274	46.27 (0.42)				
Cultural groups											
Non-participation	4,301	46.67 (0.20)			0.051	5,294	46.19 (0.20)	*	*	0.003	*p* = 0.540
Very/rather obligatory	132	45.99 (0.58)				205	46.41 (0.48)				
Rather voluntary	304	47.49 (0.41)				673	46.88 (0.31)	*			
Very voluntary	357	47.12 (0.39)				773	46.90 (0.30)		*		

The data in this table are adjusted for age, family size, BMI, comorbidities, smoking, alcohol intake, depression and cognitive functioning

^a^The total N varies slightly between each group as a result of missing data

^b^This is by use of an analysis of covariance (ANCOVA) for differences in mean scores

^*^ *P*<0.05 with the Bonferroni correction method

Table 5 shows the results on the association between the autonomy of SP and mental health. Among males, the autonomy of participation in sports groups and hobby clubs differed significantly in the MCS, and very voluntary participation had significantly better MCS than not only non-participation, but also obligatory participation. Among females, the autonomy of participation in all groups differed significantly in the MCS, and very voluntary participation in all groups except senior citizens’ clubs showed significantly better MCS than not only non-participation, but also obligatory participation. Obligatory participation in senior citizens’ clubs had significantly poorer MCS than very voluntary participation, or than rather voluntary participation. In particular, females with obligatory participation in sports groups had significantly poorer MCS than non-participation.

**Table 5:** Adjusted mean (standard error) for the Mental Health Component Summary Score for the type and autonomy of social participation, stratified by gender

	Males						Females								Interaction
	N^a^	Mean (SE)				*P* ^b^	N^a^	Mean (SE)						*P* ^b^	
Volunteer groups															
Non-participation	3,995	48.44 (0.17)				0.196	5,568	47.65 (0.17)	*					<0.001	*p* = 0.029
Very/rather obligatory	341	47.83 (0.33)					298	47.13 (0.36)		*					
Rather voluntary	379	48.11 (0.33)					567	48.13 (0.29)							
Very voluntary	372	48.43 (0.33)					522	48.68 (0.30)	*	*					
Sports groups															
Non-participation	3,627	48.37 (0.17)	*			<0.001	4,654	47.58 (0.18)	*	*				<0.001	*p* = 0.937
Very/rather obligatory	144	47.26 (0.48)		*			158	46.37 (0.47)	*		*	*			
Rather voluntary	493	48.29 (0.30)			*		757	47.79 (0.26)			*		*		
Very voluntary	811	49.20 (0.26)	*	*	*		1,361	48.60 (0.23)		*		*	*		
Hobby clubs															
Non-participation	3,230	48.34 (0.17)	*			0.009	3,674	47.58 (0.18)	*					<0.001	*p* = 0.193
Very/rather obligatory	144	47.59 (0.48)		*			173	46.37 (0.47)		*					
Rather voluntary	720	48.40 (0.26)					1,122	47.79 (0.26)			*				
Very voluntary	983	48.91 (0.24)	*	*			1,962	48.60 (0.23)	*	*	*				
Senior citizens’ clubs															
Non-participation	4,531	48.39 (0.17)				0.218	6,350	47.70 (0.17)						0.009	*p* = 0.801
Very/rather obligatory	298	47.88 (0.36)					302	47.05 (0.36)	*	*					
Rather voluntary	175	48.74 (0.44)					204	48.43 (0.42)	*						
Very voluntary	85	49.03 (0.63)					91	48.86 (0.61)		*					
Neighborhood community associations															
Non-participation	2,557	48.33 (0.18)				0.029	3,169	47.57 (0.18)	*	*				<0.001	*p* = 0.449
Very/rather obligatory	1,692	48.30 (0.20)					2,623	47.59 (0.20)			*	*			
Rather voluntary	566	48.82 (0.28)					869	48.27 (0.25)	*		*				
Very voluntary	257	49.15 (0.38)					274	49.28 (0.38)		*		*			
Cultural groups															
Non-participation	4,301	48.41 (0.17)				0.483	5,294	47.67 (0.17)	*					0.001	*p* = 0.331
Very/rather obligatory	132	48.10 (0.50)					205	46.96 (0.42)		*					
Rather voluntary	304	48.02 (0.36)					673	47.73 (0.28)							
Very voluntary	357	48.62 (0.34)					773	48.43 (0.27)	*	*					

The data in this table are adjusted for age, family size, BMI, comorbidities, smoking, alcohol intake, depression and cognitive functioning

^a^The total N varies slightly between each group as a result of missing data

^b^This is by use of an analysis of covariance (ANCOVA) for differences in mean scores

^*^ *P*<0.05 with the Bonferroni correction method

Table 6 shows the results on the association between the number of social groups and physical and mental health. Among males, those who participated in at least one group had significantly better PCS than those with non-participation, while those who participated in two or more groups did not have significantly different MCS than those with non-participation. Among females, subjects who participated in more social groups were less likely to have poor physical and mental health.

**Table 6:** Adjusted mean (standard error) for the Physical and Mental Health Component Summary Scores for the number of social groups, stratified by gender

	Males					*P* ^a^	Females							*P* ^a^	Interaction
	N	Mean (SE)					N	Mean (SE)							
Physical Health Component Summary Score															
None	1,680	46.31 (0.22)	*	*	*	0.002	1,825	45.70 (0.22)	*	*	*			<0.001	*p* = 0.080
1	1,428	47.02 (0.24)	*				1,848	46.32 (0.23)	*			*			
2	959	47.08 (0.27)		*			1,470	46.53 (0.25)		*			*		
≥3	1,059	47.11 (0.28)			*		1,863	47.24 (0.25)			*	*	*		
Mental Health Component Summary Score															
None	1,680	48.18 (0.19)	*			0.024	1,825	47.26 (0.20)	*	*	*			<0.001	*p* = 0.038
1	1,428	48.75 (0.21)	*				1,848	47.82 (0.21)	*			*			
2	959	48.30 (0.24)					1,470	48.10 (0.22)		*					
≥3	1,059	48.57 (0.24)					1,863	48.34 (0.22)			*	*			

The data in this table are adjusted for age, family size, BMI, comorbidities, smoking, alcohol intake, depression and cognitive functioning

^a^This is by use of an analysis of covariance (ANCOVA) for differences in mean scores

^*^ *P*<0.05 with the Bonferroni correction method

Regarding the effect modification by gender (shown in the right edge of Tables 2, 3, 4 and 5), significant interactions between SP and gender were observed in the frequency of participation in neighborhood community associations with the PCS (Table 2), the autonomy of participation in neighborhood community associations with the PCS (Table 4), and the autonomy of participation in volunteer groups with the MCS (Table 5), showing that females are more likely to benefit from participation in neighborhood community associations or volunteer groups.

## Discussion

This study focused on the types and frequency of SP and its autonomy, and evaluated the associations of SP with participants’ health from both physical and mental aspects. The results reconfirmed those of our previous study [20]; autonomy rather than frequency of participation in social groups showed a stronger relationship with health among community-dwelling elderly, and the positive influence was greater in females than males. We revealed that obligatory participation in some types of groups (i.e., sports groups and hobby clubs among males, and all groups among females) had a poorer MCS than voluntary participation, and obligatory participation in sports groups among females had a poorer MCS than non-participation.

Although many prior studies have indicated that SP plays a beneficial role in maintaining the mental health of older adults [4, 5, 18, 31–33], its negative effect on mental health has rarely been reported. While Mitchell and LaGory [15] reported a negative association between SP and mental health, a study that was conducted in a specific study area (i.e., an impoverished community), had a small sample size (*n* = 222) and younger study population (average age 49.5 years), and failed to evaluate the frequency and autonomy of SP. In prior studies surveying the influence of SP on the mental health of community-dwelling elderly people, researchers assessed the number of participating social groups [5] or the number of hours spent in multiple social activities [31, 33], reporting that higher SP levels were positively associated with mental health [5, 33]. Furthermore, a prospective study of older Taiwanese adults evaluated the continuity of participating in at least one social group out of 6, reporting that sustained SP had a beneficial impact on mental health [4]. We also set the number of participating social groups as a comprehensive index and evaluated the influence of SP on health, finding that females who engaged in more social groups were significantly less likely to report poor mental health. This result is in agreement with previous studies [4, 5, 33]. However, in our study, obligatory participation had significantly poorer MCS than voluntary participation, and occasionally, obligatory participation had significantly poorer MCS than non-participation. The results of the present study suggest that obligatory participation had a negative influence on mental health, and that evaluating SP with a comprehensive index is not sufficient to evaluate its influences on mental health.

Networking through SP may provide not only social support and social integration but also negative interactions. These negative aspects of social networks may act as psychological stressors [7]. Psychological stress can lead to physiological responses such as elevation of neuroendocrine function and immune function suppression, increasing the risk for poor health [34]. Regarding the mechanism of obligatory SP providing negative effects on mental health, obligatory SP may be a psychological burden on participants. It promotes psychological distress over positive psychological states, leading to mental health aggravation [9]. Additionally, when SP is obligatory, participants do not build strong connections with other people and do not have a cooperative spirit, meaning that SP may not have a stress-buffering effect [7]. However, in this study, only females with obligatory participation had significantly better PCS than non-participation, and the beneficial effect of frequency and autonomy of participation on PCS was stronger in females than in males. One possible explanation of these findings is that even though females do SP reluctantly, participation in neighborhood community associations can form close connections with neighbors, and these community-based ties may have a favorable effect on physical health of females.

Regarding gender differences in the effects of participation in volunteer groups on health, females had more mental health benefits from voluntary participation than males. This result may be linked to cultural background in the study area. The target area for this study, Nara Prefecture, has the lowest employment rate for females in Japan [35]. For females in Nara Prefecture who are unable to be part of society through work, voluntary participation in volunteer groups may be their best opportunity to develop a sense of social belonging and a purpose in life, and provide them with significant mental health benefits.

Regarding frequency of SP, our results are consistent with prior studies showing that frequent participation in certain types of SP, such as volunteer groups and neighborhood community associations, was not associated with positive health effect [2, 17]. As for volunteer groups, a prior study has suggested that frequent volunteering activity can produce exaggerated expectations from people receiving volunteer assistance, which may lay a burden on volunteers and offset the potential health benefits from volunteering [17]. As for neighborhood community associations, key roles, such as president, facilitator, and treasurer, are accustomed to be taken by rotation. Therefore, people who have a responsible role are likely to participate frequently. Because a prior study found that occupying a key position within a group did not offer health benefits to females [5], frequent participation in neighborhood community associations may have no positive effect on female physical and mental health. In contrast, frequent participation in sports groups and hobby clubs had a positive effect on physical and mental health, regardless of gender. Our results are consistent with a prior study reporting that frequent participation in these two groups had positive health effect on the elderly [2]. Sports practice can produce an increase in physical activity, which helps prevent age-related functional decline [36]. Moreover, sports practice can result in positive psychological states, which lead to maintain mental health, such as prevention of depression [37] and cognitive impairment [38]. It is also reported that hobby activities can protect the elderly against a decline in physical function [10]. Frequent participation in sports groups and hobby clubs can contribute to lively activity of sports and hobbies, which may prevent a decline in physical and mental function with aging, resulting in positive effect on physical and mental health of the elderly. And, as shown in this study’s results, because these two groups had more people with frequent participation than other groups, they might have an adequate statistical power for health effect of frequent participation. As for cultural groups, among females, monthly or more participation had significantly better MCS than yearly participation, but there was no difference in MCS between monthly or more participation and non-participation. This result suggests that yearly participation is inappropriate, and monthly or more participation is essential to achieve female beneficial effect of cultural groups on mental health.

Regarding autonomy of SP, females with obligatory participation in sports groups had significantly worse MCS than non-participation, but there was no difference in PCS between obligatory participation and non-participation. Because participation in sports groups, even if it is unwilling, is related to the increase in physical activity, it has no harmful effect on physical health. However, obligatory participation in sports groups has difficulty producing positive psychological status, which may generate a harmful effect on female mental health. Our results suggest that we require careful attention to encourage female elderly who do not like sports to participate in sports groups. As for senior citizens’ clubs, health nurses sometimes recommend aged people with risks to depression and cognitive decline to participate in senior citizens’ clubs. That is to say, people with obligatory participation in senior citizens’ clubs are more likely to have poor mental performance. This may lead to our results that obligatory participation in senior citizens’ clubs had a poorer MCS than voluntary participation.

Regarding cultural background in Japan, according to OECD Health Statistics [39], the country with the lowest percentage of the adult population reporting that their health as good is Korea, and the second lowest is Japan. Both countries have a strong Confucian background, a belief in the superiority of men over women, and a lower employment rate for females aged 25–54 compared to Western countries [40]. For Japanese females who have difficulty with self-fulfillment during adulthood, SP in later life can bring them an opportunity to realize their potential, which may have a greater effect than it does for males. Additionally, according to official Japanese statistics [41], as of June 2016, foreign residents represent 2.2% of the total population in Japan. And Japanese have national traits of disliking self-assertion and valuing harmony among people. Because Japanese live in such a homogenous community and consider relationships with others to be important, it is hypothesized that more Japanese cannot refuse SP recommended by acquaintances and unwillingly participate in social groups in greater numbers than Europeans and Americans.

We discuss the possibility of selection effect. In this study, the age range of the study population was set between the ages of 65 and 70. This is because people in this age group are those who are expected to be able to maintain and improve their living function with encouragement to participate in social activities. In the questionnaire survey subjected to community dwelling 65 years old or older people, around 10% had missing covariate values [3, 5, 42, 43], but in this study, it is only a few percent. Therefore, it seems there is a big advantage limiting study subjects to those between the ages of 65 and 70. However, the subjects in this study are the first baby boomers who were born after the Second World War. There have been changes in Japanese society as more people receive higher education and work in offices as well as urbanization and consumer-driven culture [44]. Because the baby boomer generation was strongly affected by these post-war changes, it is possible that they experienced different cultural and ideological phases than those who were born before the war. That may have influenced their SP and subjective-assessed health. Although there is no data to compare the baby boomer generation with those elderly born before the second world war, Annual Report on the Aging Society [44, 45] has reported that; 1) 61.0% of elderly people aged 60 and over participated in some group activities in 2013, 2) the most common groups which the elderly actively participate in are neighborhood community associations (26.7%), followed by hobby clubs (18.4%), and sport groups (18.3%), 3) the most common groups which the elderly want to participate in are hobby clubs (31.5%), followed by sport groups (29.7%), and neighborhood community associations (20.6%), 4) the ratio of the baby boomers participating in social activities is 38.7% in 2012, 5) the ratio of the baby boomers participating in each group is about 10% lower than that of elderly people aged 60 and over, but the type of groups which they participate in and wish to participate in showed no difference between the baby boomers and elderly people aged 60 and over, and 6) regarding the reasons why some choose not to participate in social groups, the highest number of the baby boomers replied “My work is very time consuming, and time is limited”. Among the analytical subjects in our study, the ratio of people participating in social groups is higher than that of general population. In our study, the study population was based on residents covered by the medical insurance system administered by A City, and did not include the beneficiaries of employer-provided health insurance. Therefore, our study population is considered to contain fewer workers than the general baby boomer population. Because the retired elderly have more free time than employed persons, they might have more active participation in social groups than the general population. Additionally, in this study, the response rate was insufficient. Because the respondents were likely to participate in more social groups than the non-respondents [46, 47], this selection bias may lead to a raise in the ratio of people participating in social groups in this study sample. In contrast, because our study population holds the potential to include persons who quit their work for health reasons, they might have more physical or mental impairments than the general population.

There are several limitations in this study. First, because this was a cross-sectional study, we cannot prove that there is any causal link between SP and physical and mental health. Because prior studies have pointed out that both poor physical and mental health can prevent community-dwelling elderly from participating in social groups [3, 5, 21, 42], there is a possibility of a reverse causation, that people with poor health are less likely to participate in social groups, or more likely to feel that SP is mandatory. Second, the independent variable (i.e., SP) and dependent variables (i.e., physical and mental health) were measured based on self-assessment; the possibility of a common method bias is high [48]. Our results may be overestimated due to this bias. Third, we cannot deny the possibility of residual confounding. For example, we lacked measures of socioeconomic status such as education and income. Because socioeconomic status affects SP as well as health [49, 50], additional adjustment for socioeconomic status may attenuate our results. Finally, our sample was from community-dwelling elderly aged 65–70 in Japan; therefore, generalization of our findings should be done with caution with regard to community-dwelling elderly 71 years and older or elderly living in institutions.

## Conclusions

The present study indicates that SP may not always be beneficial to the health of elderly people. When a participant participates in SP under an autonomous basis, it can be beneficial to mental health, but under an obligatory basis, its influences may be negative. In order to preserve good mental health in community-dwelling elderly, it may be important for SP strategies to respect the autonomy of the elderly. Future prospective studies are needed to establish a cause-and-effect relationship.

## Abbreviations

ANCOVA: Analysis of covariance
BADL: Basic activities of daily living
BMI: Body mass index
BP: Bodily pain
GH: General health
MCS: Mental health component summary score
MH: Mental health
PCS: Physical health component summary score
PF: Physical functioning
RE: Role-emotional
RP: Role-physical
SF: Social functioning
SF-8: 8-item short-form health survey
SP: Social participation
VT: Vitality
